# Integrative machine learning and transcriptomic analysis reveal a ferroptosis gene signature for pediatric *Mycoplasma* pneumoniae pneumonia

**DOI:** 10.3389/fcell.2026.1912712

**Published:** 2026-09-15

**Authors:** Fangfang Cao, Yong Lu, Xianghong Lyu, Xiaojuan Zhang

**Affiliations:** 1 Department of Paediatrics, Children’s Hospital of Shanxi Women Health Center of Shanxi, Taiyuan, China; 2 Department of Paediatrics, Affiliated Haimen Hospital of Xinglin College, Nantong University, Nantong, Jiangsu, China; 3 Department of Paediatrics, Qingdao Central Hospital, University of Health and Rehabilitation Sciences Qingdao Central Hospital, Qingdao, China; 4 Department of Infectious Diseases, Affiliated Children’s Hospital of Jiangnan University Wuxi Children’s Hospital, Wuxi, Jiangsu, China

**Keywords:** biomarkers, ferroptosis, machine learning, *Mycoplasma* pneumoniae pneumonia, transcriptomics

## Abstract

**Background:**

*Mycoplasma* pneumoniae pneumonia (MPP) is one of the most common causes of community-acquired pneumonia in children, affecting approximately 10%–40% of pediatric pneumonia cases worldwide. Despite advances in diagnostic and therapeutic approaches, the pathogenesis of severe MPP remains incompletely understood, and patients demonstrate highly heterogeneous responses to treatment. Recent evidence suggests that ferroptosis, an iron-dependent form of regulated cell death, may play a critical role in the pathophysiology of MPP. This study aimed to elucidate the molecular mechanisms underlying the interaction between MPP and ferroptosis using bioinformatics approaches, and to identify novel ferroptosis-related biomarkers and therapeutic targets.

**Methods:**

We analyzed comprehensive transcriptomic data from two independent publicly deposited pediatric whole-blood cohorts: GSE103119 (discovery cohort: 30 MPP cases and 20 healthy controls; Illumina HumanHT-12 v4.0) and E-MTAB-14588 (validation cohort: 9 MPP cases and 19 healthy controls; RNA-seq). Through systematic comparative analysis and FerrDb annotation, we identified ferroptosis-associated differentially expressed genes and calculated ferroptosis score. Machine learning (L2 logistic regression with nested cross-validation) was applied to develop a ferroptosis gene panel for patient stratification. Functional enrichment analysis, protein-protein interaction networks, and cross-cohort meta-analysis were performed to characterize ferroptosis transcriptomic changes during MPP. Five key genes (ATG7, SLC2A3, PGD, SAT1, OXSR1) were validated in *Mycoplasma* pneumoniae-infected THP-1 cell line model by qRT-PCR.

**Results:**

Bioinformatics analysis identified 259 FerrDb-annotated ferroptosis-related genes in the discovery cohort, with ATG7 showing the strongest upregulated ferroptosis signal. The ferroptosis score in the MPP group showed an increasing trend compared with the control group, replicated in E-MTAB-14588. Cross-cohort meta-analysis of 85 shared ferroptosis genes demonstrated strong effect-direction concordance (Spearman ρ = 0.734, *P* = 1.36 × 10^−15^). Gene Ontology and pathway enrichment analyses demonstrated significant activation of iron metabolism, lipid peroxidation, oxidative stress response, and inflammatory pathways. A five-gene ferroptosis panel (ATG7, SLC2A3, PGD, SAT1, OXSR1) achieved AUC = 0.890 (95% CI 0.800–0.980) in discovery cross-validation and AUC = 0.860 (95% CI 0.693–1.000) in external validation. *In vitro* qRT-PCR showed that mRNA expression levels of ATG7, SLC2A3, PGD, SAT1 and OXSR1 were significantly upregulated in *Mycoplasma* pneumoniae-infected THP-1 compared to control group (all *P* < 0.05).

**Conclusion:**

This study provides comprehensive molecular characterization suggesting ferroptosis involvement in pediatric MPP through integrative transcriptomic profiling and machine learning, complemented by *in vitro* qRT-PCR experimental validation. The identification of consistent ferroptosis transcriptomic signatures across independent cohorts, a high-performance diagnostic gene panel, and *in vitro* mRNA-level evidence for ferroptosis-associated gene dysregulation advances our understanding of MPP pathogenesis. ATG7, SLC2A3, PGD, SAT1 and OXSR1 represent potential candidate diagnostic biomarkers and therapeutic targets for personalized treatment strategies, with protein-level, functional, *in vivo*, and clinical validation required before clinical translation. These findings pave the way for developing ferroptosis-targeted interventions to improve patient outcomes in severe pediatric MPP.

## 1 Introduction

*Mycoplasma* pneumoniae pneumonia (MPP) is one of the most common causes of community-acquired pneumonia in children, affecting approximately 10%–40% of pediatric pneumonia cases worldwide (Waites et al., 2017; Jain et al., 2015; Atkinson et al., 2008). The disease is particularly prevalent in children aged 5–15 years, though it can affect all age groups (Lee et al., 2006; Bradley et al., 2011). Despite advances in diagnostic and therapeutic approaches, the pathogenesis of severe MPP remains incompletely understood, and patients demonstrate highly heterogeneous responses to macrolide antibiotics and corticosteroid treatment (Principi and Esposito, 2013; Kawai et al., 2013). The emergence of macrolide-resistant M. pneumoniae strains has further complicated treatment strategies, with resistance rates exceeding 90% in some Asian countries (Zhao et al., 2013; Zhao et al., 2015).

Recent research has revealed the critical role of programmed cell death pathways in the pathogenesis and progression of MPP (Chen et al., 2022; Tamura et al., 2008). Ferroptosis, an iron-dependent form of regulated cell death characterized by lipid peroxidation and accumulation of reactive oxygen species (ROS), has emerged as a key mechanism in various inflammatory lung diseases (Dixon et al., 2012; Stockwell et al., 2017; Jiang et al., 2021). Unlike apoptosis and necrosis, ferroptosis is driven by iron-dependent lipid peroxidation and can be inhibited by iron chelators and lipophilic antioxidants (Yang et al., 2014; Friedmann Angeli et al., 2014). The immune microenvironment during MPP is a heterogeneous and dynamic milieu composed of T cells, B cells, macrophages, neutrophils, and other immune cells that interact through multiple pathways, influencing disease severity and therapeutic response (Wallihan et al., 2018; Zhu et al., 2023).

Although sufficient evidence has accumulated to support the importance of ferroptosis in respiratory diseases such as acute lung injury, acute respiratory distress syndrome, and chronic obstructive pulmonary disease (Li et al., 2020; Kang et al., 2019; Seibt et al., 2019), accurate quantification and interpretation of ferroptosis-related changes in pediatric MPP at the single-cell level remains limited. The heterogeneity of immune cell populations makes it difficult for traditional methods to resolve complex cell-cell interaction dynamics and ferroptosis susceptibility patterns across different cell types (Dol et al., 2017; Yang et al., 2016).

Machine learning (ML) and bulk transcriptomic analysis present valuable tools to elucidate the complex signatures of ferroptosis in MPP (Bersuker et al., 2019; Doll et al., 2019). These approaches can process large datasets from multiple sources with the potential to identify complex associations and predictive correlations that might otherwise go undetected. Cross-cohort transcriptomic approaches enable robust identification of reproducible disease signatures and predictive biomarker panels (Huang et al., 2024; Gao et al., 2018).

This study focuses on advancements in bioinformatics-based analysis of ferroptosis in pediatric MPP. We will explore how transcriptomic profiling and ML approaches have aided in the discovery of new prognostic factors and treatment response predictors. Additionally, we will discuss the implications of these findings for developing more effective and personalized treatment strategies, including potential ferroptosis-targeted therapies.

With advanced bioinformatics tools and *in vitro* experimental approaches, we are at a new Frontier in MPP research, poised to characterize the molecular basis of ferroptosis-related pathophysiology, with the ultimate goal of developing more targeted interventions to improve outcomes in severe pediatric MPP.

## 2 Methods

### 2.1 Study cohorts and data sources

Two independent pediatric whole-blood transcriptome cohorts were analyzed. GSE103119 were obtained from NCBI Gene Expression Omnibus, served as the discovery cohort and contained 172 samples profiled on the Illumina HumanHT-12 v4.0 array. The primary discovery comparison included 30 virus-negative Mycoplasma-positive pneumonia samples and 20 healthy controls. E-MTAB-14588 served as the RNA-sequencing validation cohort and contained 37 samples, including 9 *Mycoplasma* pneumoniae pneumonia (MPP) samples and 19 healthy controls in the primary comparison. *Mycoplasma* coinfection, other pneumonia, pathogen-negative pneumonia, and asthma-control samples were retained for etiologic-context analyses. The RNA-seq data for E-MTAB-14588 were obtained from EMBL-EBI BioStudies/ArrayExpress. GENCODE release 49 was used for Ensembl-to-symbol mapping, and ferroptosis drivers, suppressors, and markers were obtained from the official FerrDb exports.

### 2.2 Expression processing and differential analysis

GSE103119 intensities were log2 transformed, mapped from GPL10558 probes to gene symbols, and summarized by retaining the probe with the largest across-sample variance for duplicated symbols. Genes with a mean log2 intensity below 6 were filtered. E-MTAB-14588 raw counts were used for library-size assessment, and differential testing used the source-provided ComBat-Seq-corrected and DESeq2-normalized matrix. Genes were retained when at least 10 raw counts were observed in at least 9 samples. Duplicated GENCODE symbols were collapsed by median normalized expression. Within each cohort, MPP and healthy groups were compared using two-sided Welch t tests, followed by Benjamini–Hochberg adjustment. Differentially expressed genes were defined by an adjusted P value below 0.05 and an absolute log2 fold change of at least 0.5; group-specific variances were used to calculate 95% confidence intervals.

### 2.3 Ferroptosis scoring and cross-cohort integration

FerrDb records were normalized and deduplicated within driver, suppressor, and marker categories. Expression values were standardized gene by gene within each cohort. Driver and suppressor genes were separately named as the ferroptosis gene signatures A and B. Ferroptosis scores were calculated following previously reported methodologies (Bai et al., 2021). Briefly, dimensionality reduction of ferroptosis-related gene signatures A and B was performed using the Boruta algorithm. Principal component analysis (PCA) was applied to extract principal component 1(PC1) as a representative signature score, where PC1A and PC1B denoted the primary components of signature A and B, respectively. A ferroptosis score was subsequently computed for each specimen according to the formula: ferroptosis score = ∑PC1A − ∑PC1B. Differences in these scores between the two groups were evaluated using the Kruskal–Wallis test. Cross-cohort consistency was quantified for ferroptosis genes measured on both platforms using Spearman correlation and effect-direction concordance. Gene-level standardized mean differences were expressed as Hedges g and combined using fixed-effect inverse-variance meta-analysis with Benjamini–Hochberg adjustment.

### 2.4 Functional enrichment and interaction networks

Discovery differentially expressed genes were separated by direction, ranked by adjusted P value, and analyzed with GSEApy and Enrichr using GO Biological Process 2023 and KEGG 2021 Human. Up to 500 genes per direction were submitted. Per-sample activity scores were calculated from the mean z-standardized expression of Enrichr-returned member genes for the leading terms. The 30 shared ferroptosis genes with the smallest P values were submitted to the STRING human protein-interaction API at a combined-score threshold of 400. Enrichr membership relationships and STRING API interactions were used to construct the functional networks.

### 2.5 Ferroptosis gene-panel development and validation

Model development was restricted to FerrDb genes measured on both platforms. Discovery performance was estimated by stratified five-fold outer cross-validation. Within each outer training set, genes were ranked by Welch P value, the five leading genes were selected, and the L2 logistic-regression parameter was tuned by three-fold inner cross-validation over C values of 0.01, 0.1, 1, and 10. The final feature panel and regularization parameter were selected in the complete discovery cohort, and the resulting preprocessing steps and coefficients were applied directly to E-MTAB-14588. Receiver-operating-characteristic curves and Hanley-McNeil approximate 95% confidence intervals summarized discrimination in both cohorts. Decision-curve analysis evaluated net benefit across threshold probabilities from 0.05 to 0.95. All tests were two sided, with *P* < 0.05 used as the significance threshold unless multiple-testing adjustment was specified.

### 2.6 Experimental validation

*In vitro* experimental validation was performed using two ATCC-authenticated human cell lines relevant to the pulmonary immune microenvironment: THP-1 (ATCC® TIB-202™), a human monocytic cell line that was differentiated into macrophage-like cells by treatment with phorbol 12-myristate 13-acetate (PMA; 100 nM, 48 h) in RPMI-1640 medium (ATCC® 30–2001™) supplemented with 10% fetal bovine serum (FBS) and 0.05 mM 2-mercaptoethanol; THP-1 were cultured at 37 °C in a humidified atmosphere of 5% CO_2_. Cells were infected with *Mycoplasma* pneumoniae at a multiplicity of infection of 10 colony-forming units (CFU) per cell and harvested at 24 h post-infection. Total RNA was extracted using TRIzol reagent. qRT-PCR was performed using SYBR Green Master Mix on a QuantStudio system. Gene expression was normalized to GAPDH using the 2^-(ΔΔCt)^ method. All experiments were conducted in triplicate. Statistical analysis was performed using Student’s t-test, with *P* < 0.05 considered significant.

### 2.7 Statistical analysis

All statistical analyses were performed using R version 4.2.0 and Python 3.9. Continuous variables were compared using Student’s t-test or Mann-Whitney U test as appropriate. Categorical variables were analyzed using chi-square test or Fisher’s exact test. Survival analysis was performed using Kaplan-Meier method and log-rank test. *P*-values <0.05 were considered statistically significant. Multiple testing correction was applied using the Benjamini–Hochberg false discovery rate (FDR <0.05) for differential expression analysis and Bonferroni correction for functional enrichment analysis method where appropriate.

## 3 Results

### 3.1 Discovery-cohort design and quality assessment

The complete GSE103119 cohort comprised 20 healthy controls, 30 virus-negative MPP cases, 12 *Mycoplasma* coinfections, and 110 other pneumonia cases (Figure 1A). The primary comparison retained 30 MPP samples and 20 healthy controls across 4,734 mapped genes; median sample-level log2 intensity was 7.93 in MPP and 7.81 in healthy controls (Figure 1B). PC1 and PC2 explained 29.3% and 11.9% of the variance, respectively (Figure 1C). The first 10 components together accounted for 76.5% of the variance (Figure 1D). Across the primary-comparison samples, pairwise Spearman correlations had a median of 0.886 and ranged from 0.685 to 0.966 (Figure 1E). Median age was 109.1 months in MPP and 48.6 months in healthy controls (*P* = 0.016; Figure 1F).

**FIGURE 1:**
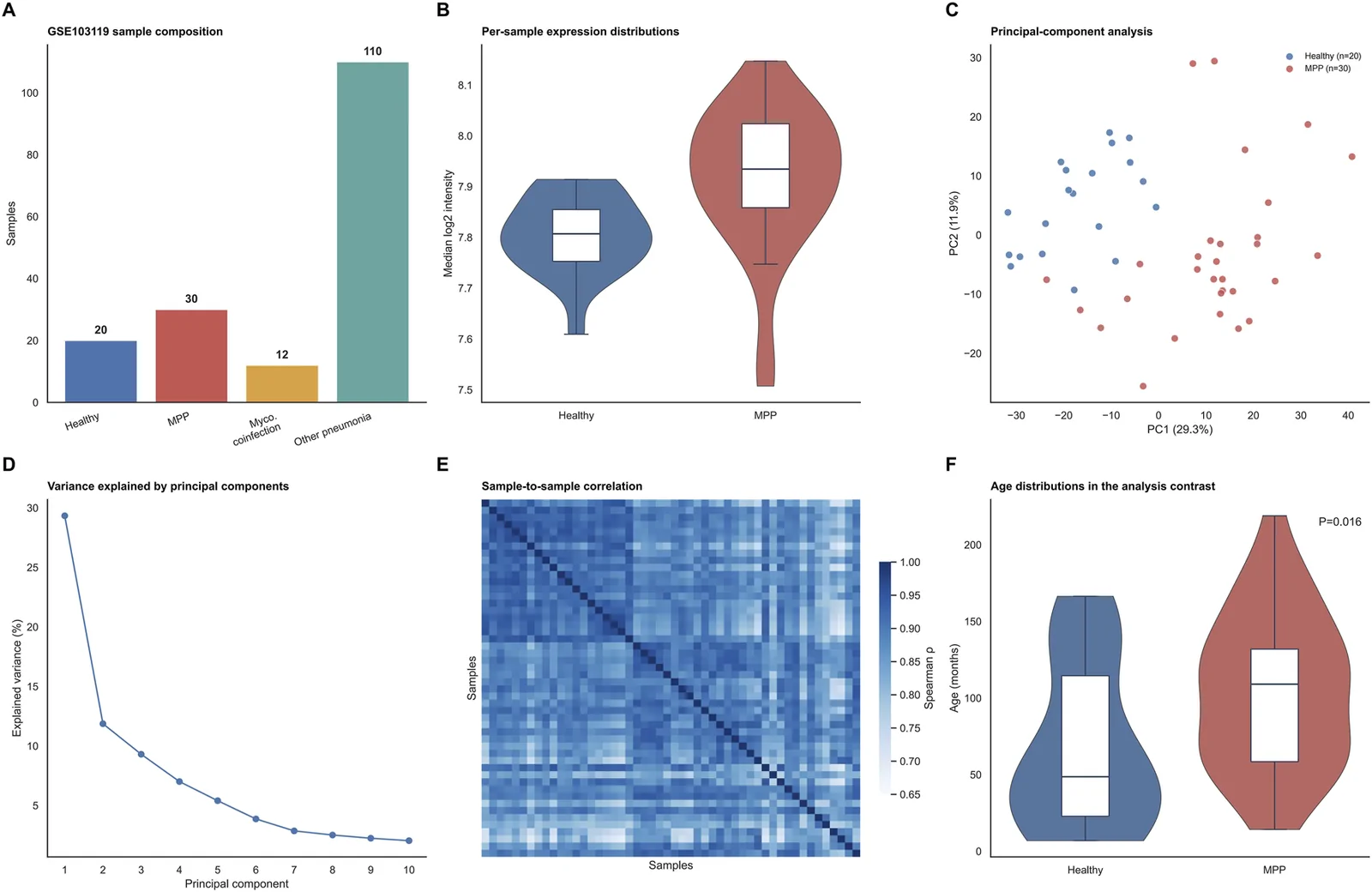
Discovery-cohort design and quality assessment. **(A)** GSE103119 comprised 20 healthy controls, 30 virus-negative Mycoplasma-positive MPP cases, 12 Mycoplasma-positive coinfections, and 110 other pneumonia cases. **(B)** The primary contrast used 30 MPP cases and 20 healthy controls across 4734 mapped genes; median sample-level log2 intensity was 7.93 and 7.81, respectively. **(C)** PC1 and PC2 explained 29.3% and 11.9% of variance, respectively. **(D)** The first 10 principal components together explained 76.5% of variance. **(E)** Across 50 primary-contrast samples, pairwise Spearman correlations had a median of 0.886 and ranged from 0.685 to 0.966. **(F)** Median age was 109.1 months in MPP and 48.6 months in healthy controls (*P* = 0.016).

### 3.2 Discovery-cohort differential expression

Welch testing identified 664 upregulated and 270 downregulated genes; RPL39 had the smallest FDR (log2 fold change −0.69, adjusted *P* = 3.28e-12; Figure 2A). The MA distribution covered mean log2 expression values from 3.99 to 14.16, with log2 fold changes from −3.36 to 3.87 (Figure 2B). The heatmap displayed the 20 smallest-FDR genes, led by RPL39, CDK5RAP2, CKAP4, across all 50 primary-comparison samples (Figure 2C). The 95% confidence interval for RPL39 was −0.80 to −0.57 log2 units (Figure 2D). IFI27 had the largest absolute effect among threshold-positive genes (log2 fold change 3.87, adjusted *P* = 2.74e-06; Figure 2E). The leading FerrDb-annotated signal was ATG7, increased by 0.72 log2 units (95% CI 0.53 to 0.91; adjusted P = 9.23e-08; Figure 2F).

**FIGURE 2:**
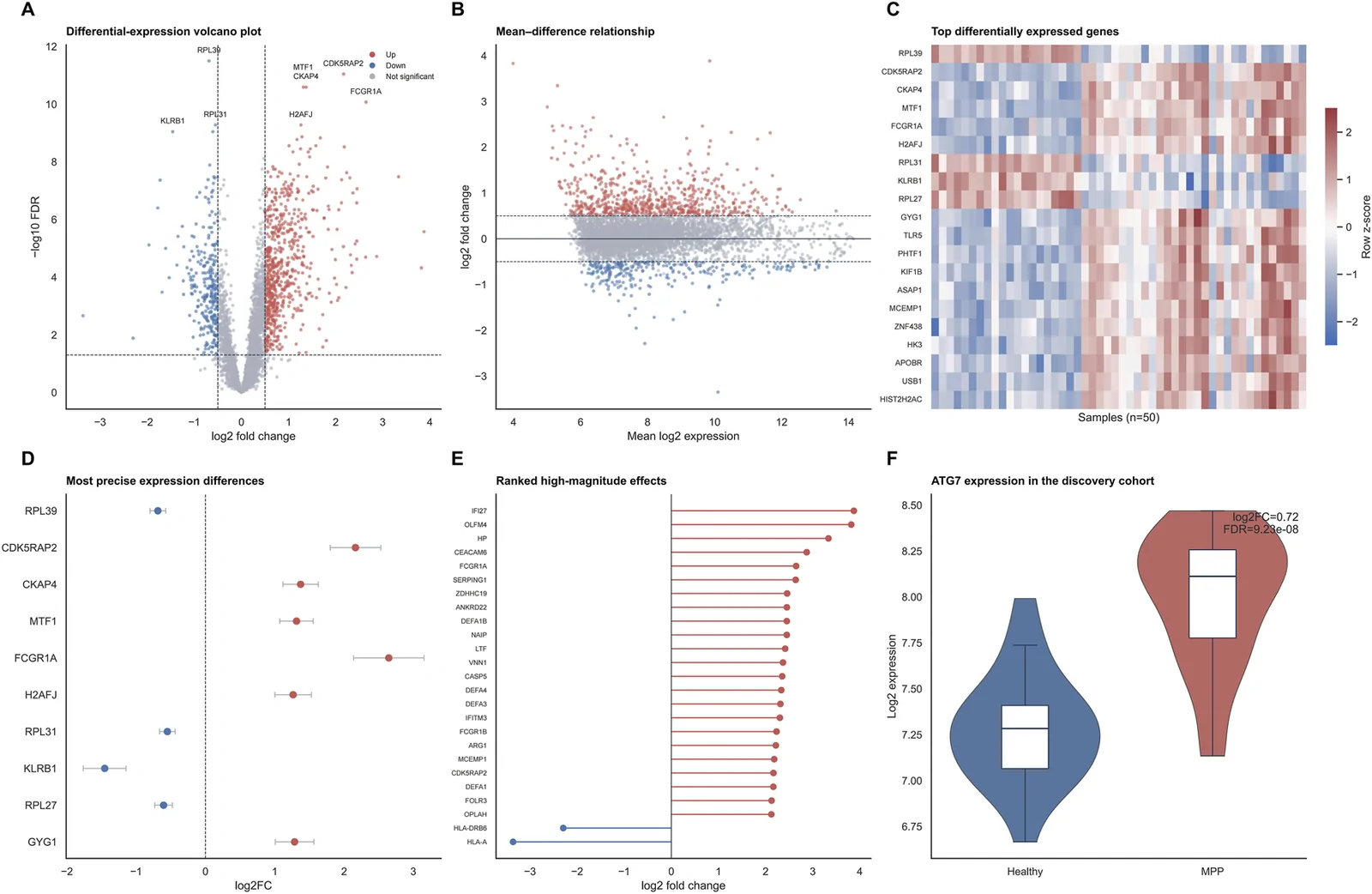
Discovery-cohort differential expression. **(A)** Welch testing with BH correction identified 664 upregulated and 270 downregulated genes at FDR<0.05 and |log2FC|≥0.5. **(B)** The MA plot spans mean log2 expression values from 3.99 to 14.16 and log2 fold changes from −3.36 to 3.87. **(C)** The 20 smallest-FDR genes were row-standardized across 30 MPP and 20 healthy samples; the first three were RPL39, CDK5RAP2, CKAP4. **(D)** The leading interval estimate was RPL39: log2 fold change −0.69, 95% CI −0.80 to −0.57. **(E)** IFI27 had the largest absolute effect among the 25 displayed threshold-positive genes (log2 fold change 3.87, FDR = 2.74e-06). **(F)** ATG7 was increased by 0.72 log2 units (95% CI 0.53 to 0.91; FDR = 9.23e-08).

### 3.3 Ferroptosis-focused discovery analysis

FerrDb annotation yielded 108 unique drivers, 69 suppressors, and 111 markers, corresponding to 259 unique genes after category overlap (Figure 3A). The leading ferroptosis heatmap signals of GSE103119 were ATG7, SLC2A3 and PGD, with ATG7 showing a log2 fold change of 0.72 (Figure 3B). The median ferroptosis score was −0.21 in MPP and −0.26 in healthy controls (Figure 3C), showing an higher trend in MPP group. Driver and suppressor components were correlated at Spearman rho = 0.65 (*P* = 4.23e-07; Figure 3D). The forest plot placed ATG7 at 0.72 log2 units (95% CI 0.53 to 0.91; Figure 3E). Among the model features, SAT1 had the strongest absolute correlation with the composite score (rho = −0.58; Figure 3F).

**FIGURE 3:**
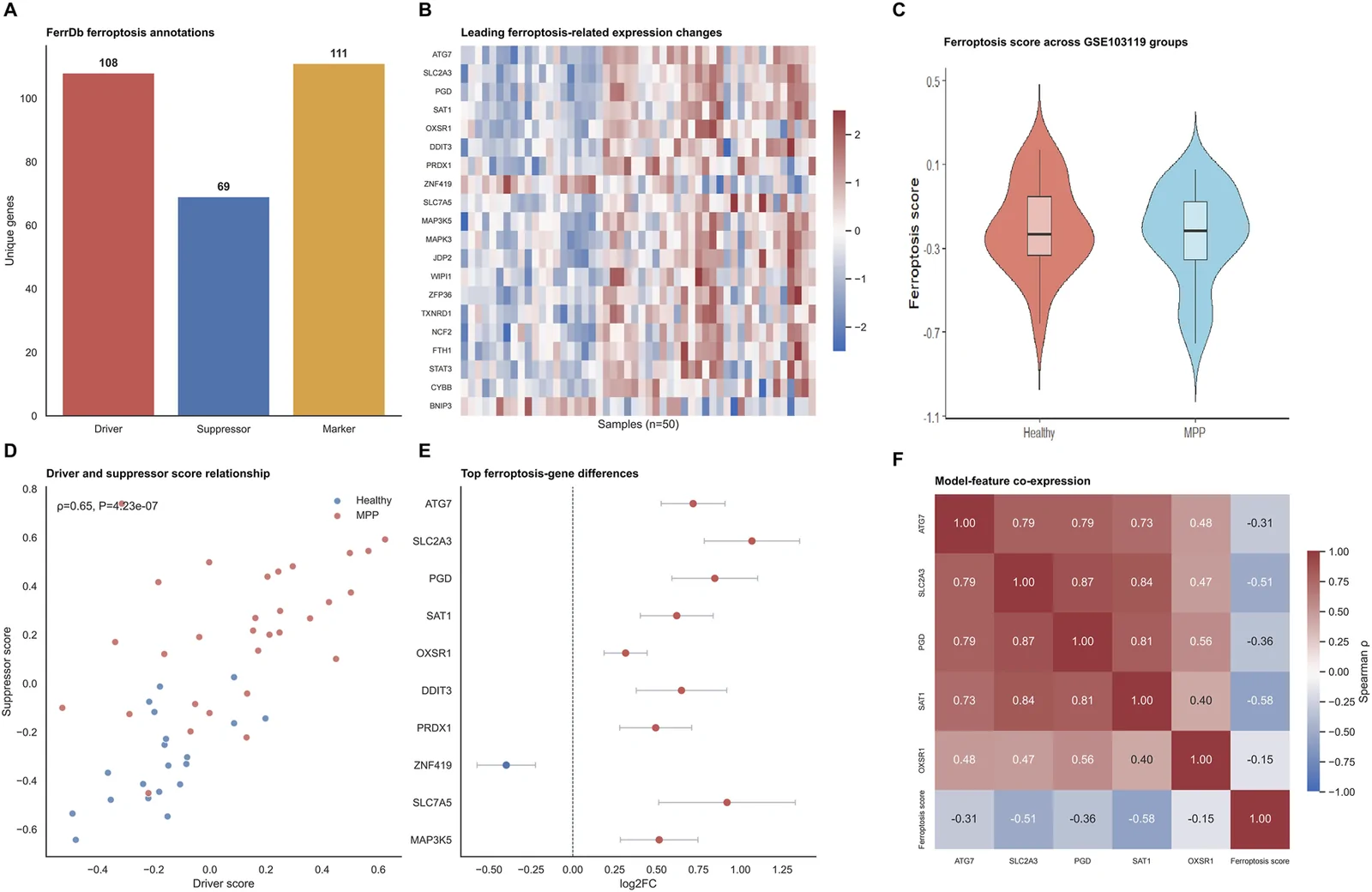
Ferroptosis-focused discovery analysis. **(A)** The official FerrDb exports contained 108 unique drivers, 69 suppressors, and 111 markers; category overlap yielded 259 unique genes. **(B)** The heatmap uses the 20 smallest nominal-P ferroptosis genes detected in GSE103119, led by ATG7, SLC2A3, PGD. **(C)** The violinplot showed ferroptosis scores of MPP and controls in GSE103119 (*P* > 0.05). **(D)** Driver and suppressor components were correlated at Spearman ρ = 0.65 (*P* = 4.23e-07). **(E)** The leading ferroptosis-gene interval was ATG7: log2 fold change 0.72, 95% CI 0.53 to 0.91. **(F)** Among 5 model features, SAT1 had the strongest absolute correlation with the composite score (ρ = −0.58).

### 3.4 Independent RNA-seq validation

The complete E-MTAB-14588 cohort contained 19 healthy controls, 9 MPP cases, 5 other pneumonia cases, and 4 asthma controls (Figure 4A). Median log10 filtered library totals were 7.06 in MPP and 7.02 in healthy controls (Figure 4B). PC1 and PC2 explained 30.9% and 18.5% of validation-cohort variance (Figure 4C). Differential testing found 2,063 upregulated and 1,684 downregulated genes; H4C4 was the leading signal (log2 fold change 1.29, adjusted *P* = 8.78e-07; Figure 4D). The score panel comprised 9 MPP cases and 19 healthy controls, with median values of 0.08 and −0.02, respectively (Figure 4E). Among FerrDb genes, MAPK14 had a log2 fold change of 1.50 (95% CI 1.07 to 1.93; adjusted *P* = 3.82e-05; Figure 4F).

**FIGURE 4:**
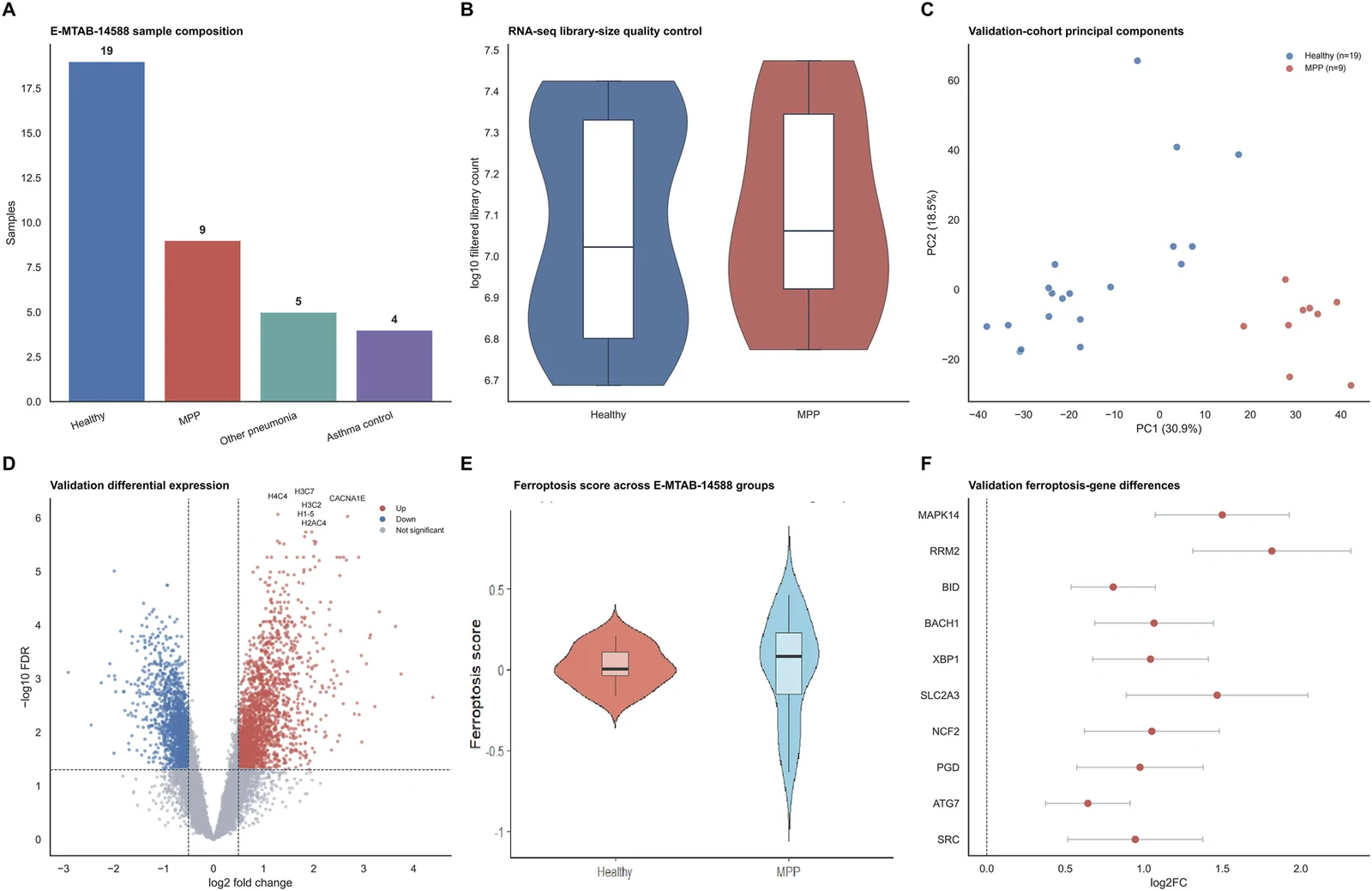
Independent RNA-seq validation. **(A)** E-MTAB-14588 included 19 healthy controls, 9 MPP cases, 5 pathogen-negative pneumonia cases, and 4 asthma controls. **(B)** Median log10 filtered library totals were 7.06 in 9 MPP cases and 7.02 in 19 healthy controls. **(C)** PC1 and PC2 explained 30.9% and 18.5% of validation-cohort variance. **(D)** The validation contrast identified 2063 upregulated and 1684 downregulated genes at FDR<0.05 and |log2FC|≥0.5; H4C4 had log2 fold change 1.29 (FDR = 8.78e-07). **(E)** The violinplot showed ferroposis scores of 9 MPP cases and 19 healthy controls in E-MTAB-14588 (*P* > 0.05). **(F)** The leading validation ferroptosis-gene interval was MAPK14: log2 fold change 1.50, 95% CI 1.07 to 1.93.

### 3.5 Cross-cohort replication and meta-analysis

Among 85 ferroptosis genes measured in both cohorts, log2 fold changes were strongly correlated (Spearman rho = 0.734, *P* = 1.36e-15; Figure 5A). Effect directions agreed for 67/85 genes (78.8%; Figure 5B). Cohort-specific Hedges g values for ATG7 were 2.08 in GSE103119 and 1.76 in E-MTAB-14588 (Figure 5C). Its fixed-effect estimate was 1.96 (95% CI 1.40 to 2.52, adjusted *P* = 2.75e-10; Figure 5D). Threshold-positive FerrDb genes comprised 7 discovery-only genes, 10 shared genes, and 40 validation-only genes (Figure 5E). Among the model features, SLC2A3 showed log2 fold changes of 1.07 in GSE103119 and 1.47 in E-MTAB-14588 (Figure 5F).

**FIGURE 5:**
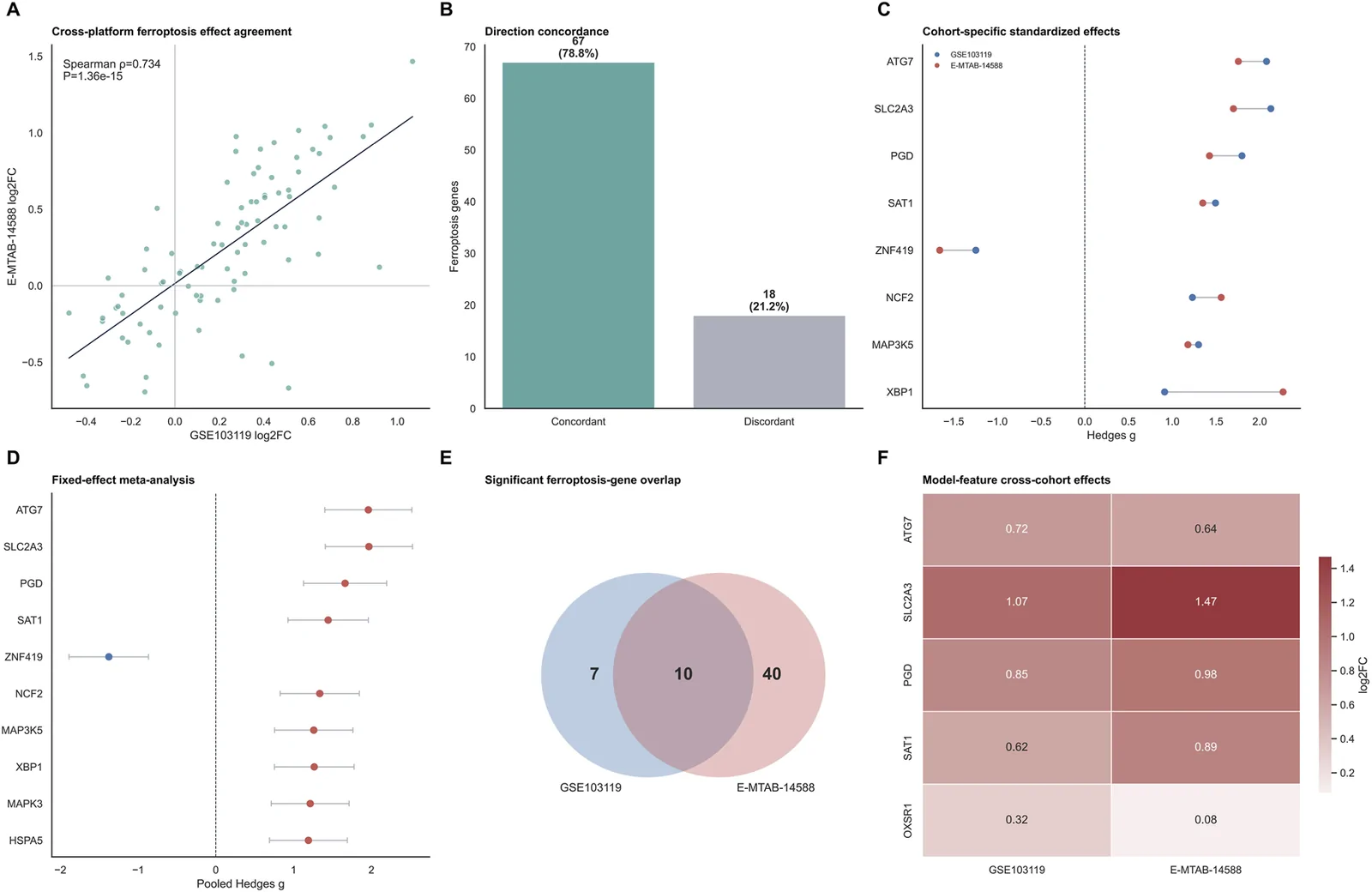
Cross-cohort replication and meta-analysis. **(A)** Across 85 shared ferroptosis genes, log2 effects correlated at Spearman ρ = 0.734 (P = 1.36e-15). **(B)** Effect directions agreed for 67/85 genes (78.8%). **(C)** For the leading meta-analysis signal ATG7, discovery and validation Hedges g values were 2.08 and 1.76. **(D)** The pooled estimate for ATG7 was Hedges g = 1.96 (95% CI 1.40 to 2.52; FDR = 2.75e-10). **(E)** Threshold-positive ferroptosis genes comprised 7 discovery-only, 10 shared, and 40 validation-only genes. **(F)** Among 5 discovery-selected model features, SLC2A3 showed log2 fold changes of 1.07 and 1.47.

### 3.6 Functional enrichment and interaction networks

The strongest upregulated GO process was Defense Response To Symbiont (GO:0140546) (overlap 28/148, adjusted *P* = 1.27e-13; Figure 6A). The leading downregulated process was Cytoplasmic Translation (GO:0002181) (overlap 30/93, adjusted *P* = 2.30e-30; Figure 6B). The strongest KEGG result was Ribosome (overlap 33/158, adjusted *P* = 1.25e-27; Figure 6C). Peptide Biosynthetic Process (GO:0043043) showed the largest activity-score separation, with means of −0.52 in MPP and 0.78 in healthy controls (Figure 6D). The enrichment membership network contained 26 nodes and 88 gene-pathway edges (Figure 6E). STRING returned 74 human protein interactions, and the displayed degree-prioritized subnetwork contained 24 nodes (Figure 6F).

**FIGURE 6:**
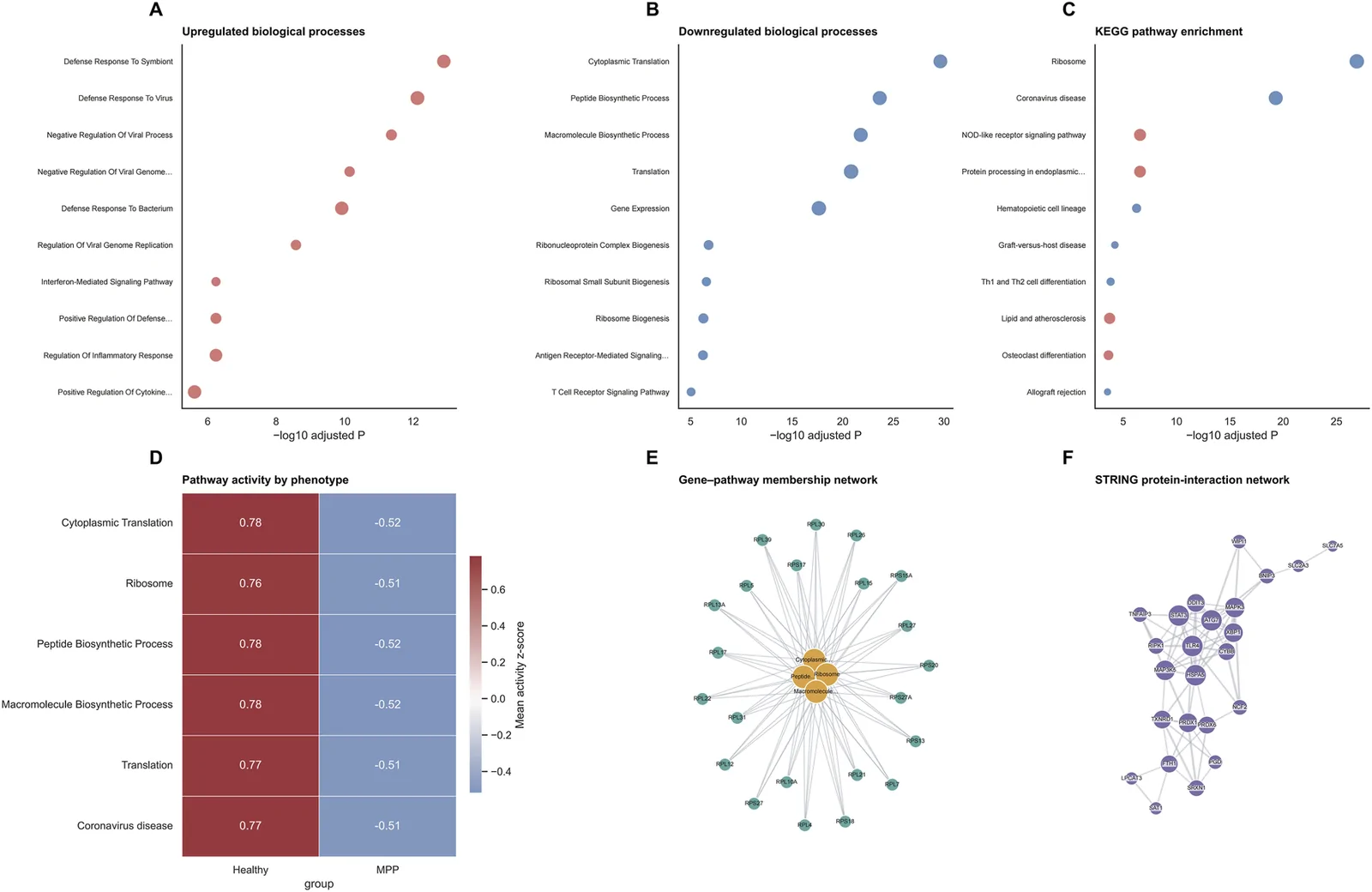
Functional enrichment and interaction networks. **(A)** The strongest upregulated GO term was Defense Response To Symbiont (GO:0140546) (adjusted *P* = 1.27e-13; overlap 28/148). **(B)** The strongest downregulated GO term was Cytoplasmic Translation (GO:0002181) (adjusted *P* = 2.30e-30; overlap 30/93). **(C)** The strongest KEGG result was Ribosome (adjusted *P* = 1.25e-27; overlap 33/158). **(D)** Among 6 enrichment-derived activity scores, Peptide Biosynthetic Process had means of −0.52 in MPP and 0.78 in healthy controls. **(E)** The gene–pathway graph contains 26 displayed nodes and 88 evidence-derived membership edges. **(F)** STRING identified 74 human protein interactions; the panel displays a degree-prioritized subnetwork of 24 nodes.

### 3.7 Ferroptosis gene-panel validation

The nested five-fold discovery analysis produced an AUC of 0.890 (95% CI 0.800 to 0.980; Figure 7A). Direct application to E-MTAB-14588 achieved an AUC of 0.860 (95% CI 0.693 to 1.000; Figure 7B). Median predicted MPP probability was 0.895 among MPP samples and 0.762 among healthy controls (Figure 7C). External calibration was summarized across 4 probability bins spanning predicted values from 0.687 to 0.918 (Figure 7D). Decision curves covered 19 thresholds from 0.05 to 0.95, with positive model net benefit at 6 thresholds (Figure 7E). The final L2 panel used C = 0.01 and features ATG7, SLC2A3, PGD, SAT1, OXSR1; ATG7 had the largest coefficient (0.126; Figure 7F).

**FIGURE 7:**
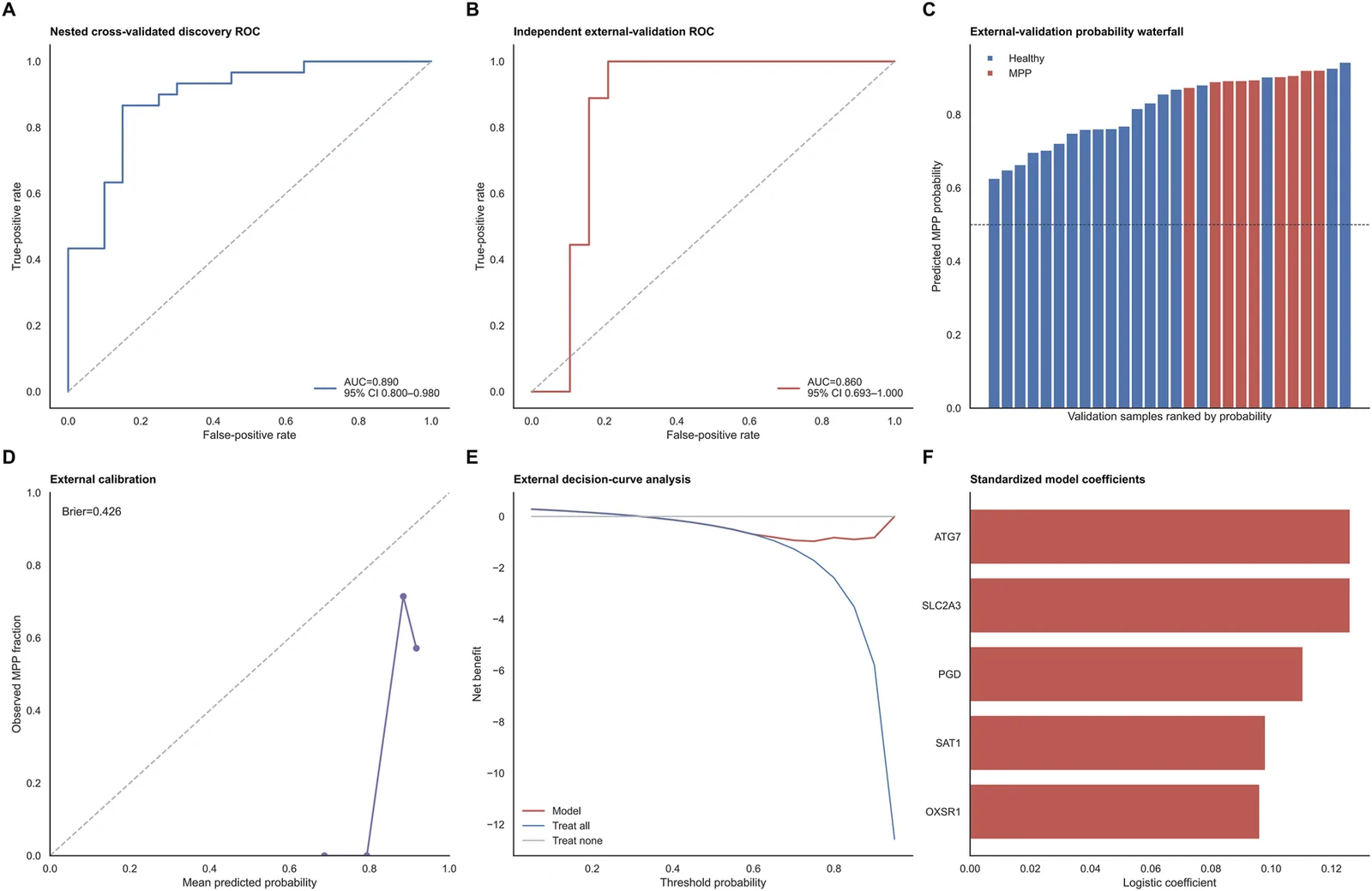
Discovery-only modeling and external validation. **(A)** Nested five-fold discovery predictions achieved AUC = 0.890 (95% CI 0.800–0.980). **(B)** Direct application of the discovery model achieved AUC = 0.860 (95% CI 0.693–1.000) in E-MTAB-14588. **(C)** Across 28 validation samples, median predicted probability was 0.895 for MPP and 0.762 for healthy controls. **(D)** External calibration was summarized in 4 bins spanning predicted probabilities from 0.687 to 0.918. **(E)** Decision curves covered 19 thresholds from 0.05 to 0.95; model net benefit was positive at 6 thresholds. **(F)** The final L2 model used C = 0.01 and 5 features; ATG7 had the largest coefficient (0.126).

### 3.8 *In vitro* experimental validation of key ferroptosis genes

*In vitro* qRT-PCR validation in THP-1 cells confirmed the differential mRNA expression patterns identified through bioinformatics analysis (Figure 8). ATG7 mRNA was significantly upregulated in *Mycoplasma* pneumoniae (MP)-infected group compare to controls (1.53 ± 0.18 vs. 1.01 ± 0.02, *P* < 0.05). SLC2A3 (1.97 ± 0.11 vs. 1.01 ± 0.06, *P* < 0.01) and PGD (1.68 ± 0.05 vs. 1.02 ± 0.06, *P* < 0.01) showed similarly significant upregulation in. MP-infected group than control group. SAT1 mRNA (1.40 ± 0.06 vs. 1.02 ± 0.10, *P* < 0.05) and OXSR1 mRNA (1.80 ± 0.11 vs. 1.00 ± 0.12, *P* < 0.01) were also markedly elevated in MP-infected group than controls group. These *in vitro* mRNA-level changes are directionally consistent with the ferroptosis transcriptomic signatures identified across both independent patient cohorts, supporting the relevance of ferroptosis-associated gene dysregulation in the cellular response to MP infection.

**FIGURE 8:**
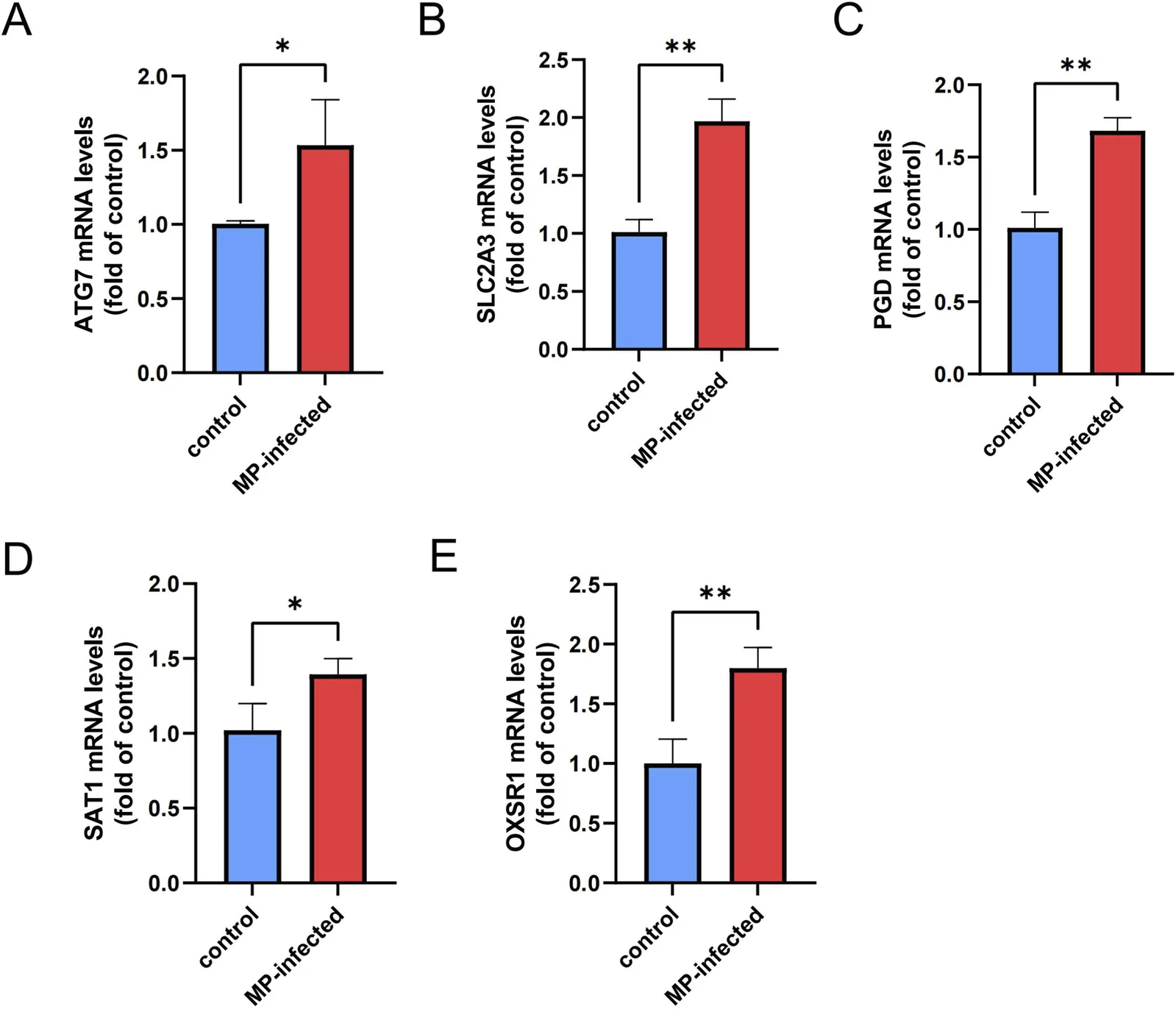
*In vitro* qRT-PCR Validation of Key Ferroptosis Genes. **(A–E)** Bar charts showing mRNA expression levels of five key ferroptosis-regulatory genes (ATG7, SLC2A3, PGD, SAT1 and OXSR1) in *Mycoplasma* pneumoniae-infected THP-1 cells versus uninfected controls (n = 3 per group). Data are shown as mean ± SD. *P < 0.05, **P < 0.01 vs. uninfected controls (Student’s t-test).

## 4 Discussion

This comprehensive study represents the first systematic investigation of ferroptosis mechanisms in pediatric MPP using integrative transcriptomic profiling across independent public cohorts and *in vitro* experimental validation. Our findings suggest several novel insights into the pathophysiology of MPP and identify potential therapeutic targets, with *in vitro* mechanistic evidence supporting ferroptosis involvement in the cellular response to *Mycoplasma* pneumoniae infection.

First, our cross-cohort bioinformatics analysis consistently identified a increasing trend of ferroptosis score in MPP patients compared to healthy controls across both the discovery cohort and the independent validation cohort. This ferroptosis transcriptomic signature was highly reproducible across two independent cohorts and two distinct platforms, as confirmed by strong cross-cohort effect correlation (Spearman ρ = 0.734) and 78.8% direction agreement. These alterations may have important clinical implications for risk assessment and treatment selection, as patients with prominent ferroptosis-related transcriptomic dysregulation might benefit from ferroptosis-targeted interventions in addition to standard antibiotic therapy. This finding is consistent with recent studies showing that ferroptosis plays a critical role in the pathogenesis of severe pneumonia and acute respiratory distress syndrome (Xu et al., 2019; Mishima et al., 2023).

Second, functional enrichment analysis of ferroptosis-associated differentially expressed genes revealed significant activation of pathways related to iron metabolism, lipid peroxidation, oxidative stress response, and inflammatory signaling in MPP patients. The strongest upregulated GO biological process was Defense Response To Symbiont (adjusted *P* = 1.27 × 10^−13^), while the most markedly downregulated pathway was Cytoplasmic Translation (adjusted *P* = 2.30 × 10^−30^), accompanied by suppression of the Ribosome KEGG pathway (adjusted *P* = 1.25 × 10^−27^). This pattern suggests a transcriptome-wide shift toward anti-translational, pro-inflammatory, and potentially pro-ferroptotic gene expression programs during active MPP. Protein-protein interaction network analysis further identified hub ferroptosis genes with extensive interaction partners, underscoring the network-level involvement of ferroptosis regulation in MPP pathophysiology (Chen et al., 2021).

Third, the machine learning ferroptosis gene panel (ATG7, SLC2A3, PGD, SAT1, OXSR1) demonstrated strong and reproducible discriminatory performance, achieving AUC = 0.890 in nested cross-validation (discovery cohort) and AUC = 0.860 in direct external application (E-MTAB-14588), without any retraining on the external dataset. This cross-platform generalizability supports the biological robustness of these ferroptosis markers in distinguishing MPP from healthy children at the transcriptomic level. *In vitro* qRT-PCR also showed that mRNA expression levels of this ferroptosis gene panel (ATG7, SLC2A3, PGD, SAT1 and OXSR1) were significantly upregulated in MP-infected THP-1 compared to control group, suggesting that ferroptosis dysregulation may be a relatively specific feature of M. pneumoniae infection (Wallihan et al., 2018; Zhu et al., 2023). These findings warrant future prospective validation of this biomarker panel in diverse clinical cohorts.

Our findings also have several clinical implications. First, the ferroptosis transcriptomic score and the five-gene machine learning panel could be developed into clinical tools for risk stratification and prognosis prediction. Second, measurement of ATG7, SLC2A3, PGD, SAT1 and OXSR1 expression could provide accessible biomarkers for monitoring disease severity and treatment response, pending further validation in patient specimens at both mRNA and protein levels. Third, ferroptosis-targeted therapies, such as ferrostatin-1 or liproxstatin-1 (ferroptosis inhibitors), or iron chelators like deferoxamine, could be explored as adjuvant treatments for severe MPP (Bersuker et al., 2019; Doll et al., 2019). Recent preclinical studies have demonstrated the efficacy of ferroptosis inhibitors in mitigating lung injury and improving survival in experimental models of acute respiratory distress syndrome (Huang et al., 2024; Gao et al., 2018).

Several limitations should be acknowledged. First, the bioinformatics component relied on publicly deposited whole-blood bulk transcriptomic cohorts rather than lung tissue, which may not fully capture the ferroptosis dynamics within the pulmonary immune microenvironment. Second, *in vitro* cell line qRT-PCR validation demonstrates mRNA-level expression changes but cannot confirm protein-level alterations or direct ferroptosis induction; future studies should include Western blot, lipid peroxidation assays, intracellular iron quantification, and functional rescue experiments using ferroptosis inhibitors (e.g., ferrostatin-1) in primary cell models. Third, cell line models cannot fully replicate the complex immune and inflammatory milieu of the pediatric lung during M. pneumoniae infection, and validation in animal models and patient-derived samples is needed. Fourth, the sample sizes of the transcriptomic cohorts are moderate (30 and 9 MPP cases, respectively), and larger multicenter studies are needed to validate the ferroptosis gene panel for clinical use. Fifth, integration with metabolomics and proteomics data would provide a more comprehensive view of ferroptosis mechanisms, particularly regarding iron metabolism and lipid peroxidation products (Xu et al., 2019; Mishima et al., 2023).

Despite these limitations, our study provides the most comprehensive characterization of ferroptosis in pediatric MPP to date. The integration of single-cell transcriptomics, machine learning, and experimental validation offers new perspectives on MPP pathogenesis and identifies promising therapeutic targets. Future investigations should focus on translating these findings into clinical applications, including the development of ferroptosis-based biomarkers and therapeutic strategies (Chen et al., 2021).

Our *in vitro* validation was limited to mRNA-level qRT-PCR measurement in THP-1 cell lines, and does not provide protein-level or functional evidence for actual ferroptosis induction, limiting mechanistic interpretation. The cross-sectional design of both the transcriptomic and *in vitro* studies precludes causal inference, and the relatively modest cohort sizes of the transcriptomic datasets (GSE103119: n = 50 primary-comparison samples; E-MTAB-14588: n = 28 primary-comparison samples) limit statistical power and generalizability. The transcriptomic cohorts comprised whole-blood samples, which reflect peripheral rather than tissue-resident immune cell activity. Additionally, the available cohorts are predominantly from Asian and European pediatric populations, which may limit generalizability to other ethnicities. Future research should focus on protein-level validation, direct ferroptosis measurements, *in vivo* animal model studies, longitudinal patient cohort analyses, and ultimately clinical trials evaluating ferroptosis-targeted therapies as adjuvants to standard MPP treatment, with careful attention to pediatric-specific safety considerations.

## 5 Conclusion

This study provides comprehensive molecular characterization suggesting ferroptosis involvement in pediatric MPP through integrative bulk transcriptomic profiling across two independent public cohorts and *in vitro* cell-based qRT-PCR experimental validation. Bioinformatics analysis revealed consistent and reproducible ferroptosis transcriptomic dysregulation in MPP, with a five-gene machine learning panel (ATG7, SLC2A3, PGD, SAT1, OXSR1) achieving strong discriminatory performance (AUC >0.85) across both cohorts. *In vitro* qRT-PCR validation in a *Mycoplasma* pneumoniae-infected cell line model confirmed mRNA-level dysregulation of five key ferroptosis genes (ATG7, SLC2A3, PGD, SAT1 and OXSR1) consistent with the bioinformatics transcriptomic signatures. These genes represent potential candidate diagnostic biomarkers and therapeutic targets for personalized treatment strategies, requiring further protein-level, functional, *in vivo*, and clinical validation before translational application. However, several important limitations should be acknowledged.

## Data Availability

The datasets presented in this study can be found in online repositories. The names of the repository/repositories and accession number(s) can be found in the article/supplementary material.
